# Asymptomatic Intracorneal Graphite Deposits following Graphite Pencil Injury

**DOI:** 10.1155/2012/720201

**Published:** 2012-04-17

**Authors:** Swetha Sara Philip, Deepa John, Sheeja Susan John

**Affiliations:** Department of Ophthalmology, Christian Medical College and Hospital, Tamil Nadu, Vellore 632001, India

## Abstract

Reports of graphite pencil lead injuries to the eye are rare. Although graphite is considered to remain inert in the eye, it has been known to cause severe inflammation and damage to ocular structures. We report a case of a 12-year-old girl with intracorneal graphite foreign bodies following a graphite pencil injury.

## 1. Introduction

Ocular injuries with graphite pencil lead are rare. There is only one case report of intracorneal graphite foreign bodies following graphite pencil lead injury in literature to date [1]. We report a case of a 12-year-old girl with intracorneal graphite foreign bodies, which remained inert in the eye for the past three years.

## 2. Case Report

**Figure 1:**
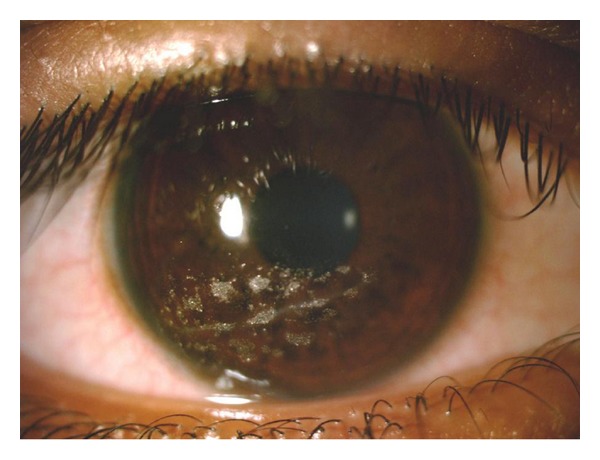
Graphite particles seen within the corneal stroma of the right eye.

A 12-year-old girl came to our outpatient clinic for a routine ophthalmological checkup. She had been using glasses for the past three years. She did not complain of any other problems in her eyes. On examination, her best corrected visual acuity (BCVA) was found to be 6/6 J1 with myopic astigmatism in both eyes. Examination of the anterior segment of the right eye showed a linear anterior stromal corneal scar, which was located at the inferior pupillary margin. The scar measured about 4.5 mm and was studded with refractile particles along its entire length (Figure 1). There was no evidence of any damage to any intraocular structures. There was no evidence of past or present inflammation in the eye. The left eye was normal.

When questioned regarding any past ocular injury, the parents told us that the patient had been accidentally poked in the right eye with a graphite pencil about three years ago. There was mild redness and pain in the eye soon after the injury. This was treated with some topical antibiotics and lubricants. The symptoms resolved with this treatment and she has been entirely asymptomatic to date.

Since there was no evidence of active inflammation or progressive damage to the ocular structures, we decided against any further intervention. The patient is currently on followup.

## 3. Discussion

 Graphite pencils are made of graphite and clay, mixed with animal oils and fats, with a wooden surround [2]. Although graphite pencils are universally used in classrooms and homes across the world by young children, there are not many reports of injuries to the eye and adnexa with these pencils [3–8]. In the reported cases of ocular injury with graphite pencil lead, the commonly injured sites are the eyelids and orbit [3, 4]. This could be due to the fact that when an object is brought close to the eye, there is sudden closure of both eyes as part of the protective “menace reflex.” Consequently, there is less chance of injury to the eye itself.

There are only nine reports of injury to the eye with graphite pencil lead in literature [4–10]. Graphite is generally known to remain inert in the eye [4]. However, there have been reports of severe inflammatory reaction and endophthalmitis following graphite pencil lead injury [5]. It has been suggested that the other components of the pencil like wood or aluminium may have been the cause of severe ocular inflammatory reaction in these cases [5]. A case in which a graphite foreign body in the conjunctiva simulated a melanoma, resulting in surgical intervention, has also been reported [7]. There has only been one report of intracorneal graphite foreign bodies in literature [1]. As in our case, the patient was totally asymptomatic and had good visual acuity in the involved eye.

There is paucity of data regarding ocular injuries with graphite pencil lead. It is possible that many such injuries go unnoticed and unreported as the patients are often totally asymptomatic. However, in view of the fact that graphite pencil lead injuries have on rare occasions, been reported to cause severe ocular inflammatory reaction and endophthalmitis, resulting in poor vision, it is imperative that all such cases be reported.
